# Earring ingestion in a twelve-month-old infant

**DOI:** 10.11604/pamj.2025.50.94.47381

**Published:** 2025-04-08

**Authors:** Leila Debono, Lamia Karboubi

**Affiliations:** 1 Pediatric Medical Emergency, Children's Hospital of Rabat, Rabat, Morocco,; 2 Faculty of Medicine and Pharmacy of Rabat, Mohamed V University in Rabat, Rabat, Morocco

**Keywords:** Ingestion, earring, foreign body, infant

## Image in medicine

A 12-month-old girl, with no prior history, ingested a gold-plated earring, which immediately triggered vomiting. The patient was admitted to the pediatric medical emergency department at Rabat Children's Hospital 16 hours after ingestion, with hypersalivation and no other associated signs. A frontal chest X-ray was performed, revealing a radiopaque foreign body in the upper third of the esophagus. A gastrointestinal endoscopy under sedation was performed with the goal of extraction. The pediatric fiberscope was inserted up to 10 cm, and the foreign body was found to be covered with food debris at this level. Extraction using fine forceps was successful on the first attempt. Post-extraction exploration revealed superficial erosions of the surrounding mucosa. The infant was discharged from the hospital after 12 hours of monitoring following the procedure. She was given oral corticosteroid therapy at 2 mg/kg/day, with omeprazole 1 mg/kg and amoxicillin clavulanic acid at 50 mg/kg/day for 7 days. At the last follow-up after 1 month, no symptoms had been reported by the mother, and the clinical examination was normal.

**Figure 1:**
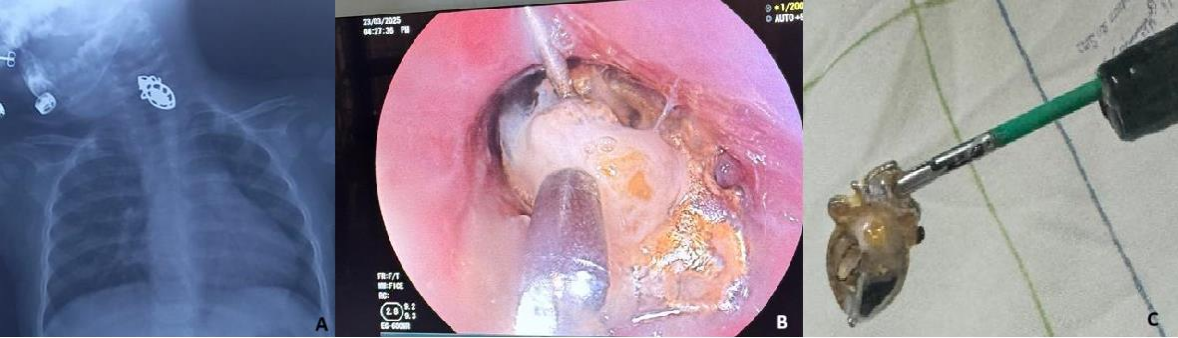
A) chest X-ray showing the foreign body in the upper third of the esophagus; B) endoscopic image showing the extraction of the earring covered in food debris by forceps; C) image of the extracted earring

